# 2100 TL1 team approach to social and genetic determinants of nocturnal blood pressure

**DOI:** 10.1017/cts.2018.231

**Published:** 2018-11-21

**Authors:** Leanne Dumeny, Chu Hsiao, Larisa H. Cavallari, Connie J. Mulligan, Wayne T. McCormack

**Affiliations:** University of Florida

## Abstract

OBJECTIVES/SPECIFIC AIMS: The TL1 Team approach aims to train translational investigators capable of tackling complex and multifaceted diseases, such as hypertension, by beginning multidisciplinary, team-based training early in their graduate programs. METHODS/STUDY POPULATION: Leanne Dumeny is a graduate student in Genetics and Genomics studying how pharmacogenomics can be applied to improve clinical care and cardiovascular outcomes. Chu Hsiao is a graduate student in Anthropology studying how sociocultural experiences become biologically embodied. Both are in the Ph.D. phase of M.D.-Ph.D. training. Joining the seemingly disparate but complementary fields of anthropology and genomics facilitates understanding of the intersection between socially driven experiences and genetics on nocturnal blood pressure. Understanding both social determinants, such as racial discrimination, and biological determinants, such as genetics, is important because an interplay of gene-environment interactions influences many complex diseases. Rarely can 1 individual, or 1 discipline, tackle all the perspectives necessary to answer these types of complex questions. The TL1 Team curriculum teaches students to navigate the spectrum of translational research as a team, reflect on disciplinary limitations, and embrace collaborative research. RESULTS/ANTICIPATED RESULTS: This team project will investigate the relationship between racial discrimination and genetics using a large epidemiological cohort of African Americans in Mississippi. The data request application is currently under review. By the project’s end, the team anticipates their investigation will reveal novel associations between racial discrimination, genetic polymorphisms, and nocturnal blood pressure measurements. The investigators will have gained experience obtaining and analyzing large external data sets, working in diverse team settings, collaborating across state-lines, and publishing articles. Through this team approach, the students will also understand the barriers to working in multidisciplinary groups, and develop a foundation for approaching future collaborations. DISCUSSION/SIGNIFICANCE OF IMPACT: By joining anthropology with genomics, it becomes possible to understand the intersection between socially driven experiences of racial discrimination and genetics on nocturnal blood pressure. The successful training of this first cohort of team-applicants to the TL1 funding mechanism can impact how graduate education will be structured and could reframe graduate education to emphasize a team-based approach.

